# Contributions of Europeans to Xenotransplantation Research: 2. Pig Islet and Cell Xenotransplantation

**DOI:** 10.3389/ti.2025.14143

**Published:** 2025-04-17

**Authors:** Rita Bottino, Krish Vasudev, Zuzanna Iwanczyk, Emanuele Cozzi, David K. C. Cooper

**Affiliations:** ^1^ Imagine Islet Center, Imagine Pharma, Pittsburgh, PA, United States; ^2^ Department of Surgery, Center for Transplantation Sciences, Massachusetts General Hospital/Harvard Medical School, Boston, MA, United States; ^3^ Transplantation Immunology Unit, University of Padua Hospital, Padua, Italy

**Keywords:** Europeans, genetic engineering, pancreatic islets, pig, USA, xenotransplantation

## Abstract

Pig islet xenotransplantation in nonhuman primates (NHPs) has made considerable progress during the past 30 years, and European scientists in both Europe and the USA have contributed to this progress. At times, there have been, or are, active research programs in Sweden, Germany, Belgium, and the USA. The first clinical experiments of wild-type (i.e., genetically-unmodified) pig islet xenotransplantation were carried out by Groth and his colleagues in Stockholm in 1994, but without significant success. Hering’s group in Minneapolis was the first to report prolonged survival of wild-type pig islets in NHPs in 2006, and the first report of insulin-independence for >12 months was by a “European” research team at the University of Pittsburgh in 2009. Recent progress has been slow, in part through a lack of funding, but recent advances in pig organ xenotransplantation suggest that pig islet xenotransplantation is poised for clinical experiments in the near future. In addition, there have been encouraging experimental studies of pig neural cell injections into the brains of monkeys with a pharmacologically-induced Parkinson’s disease.

## Introduction

The contributions of Europeans to our understanding of pancreatic islets go back to their identification in 1869 by a German medical student, Paul Langerhans (1847–1888), although he did not know their function. The first attempt to transplant pancreatic tissue was in 1916 by the British surgeon, Frederick Pybus (1893–1975). We also need to consider the very controversial work in the 1920s and 1930s of Serge Voronoff (1866–1951), a Russian émigré in Paris who, although his work on testicular transplants was misguided, at least introduced the concept of transplanting xenogeneic cells that would provide replacement of native cells that had lost their function, e.g., insulin-producing islets.

The transplantation of porcine pancreatic islets for Type 1 diabetic patients has the potential to replace the current “gold standard” treatment of pancreatic allotransplantation. Many European researchers have contributed to advancing this area of xenotransplantation towards clinical reality, with the potential to impact millions of patients living with diabetes globally – particularly so as the number of patients drastically out-numbers pancreas donors for allotransplantation, and frequently islets from two or more human donors are required for one recipient. Approaches to balance the impact of diabetes and the risks of immunosuppression have varied between (i) focusing on combined islet-kidney transplants (where immunosuppressive therapy is administered for protection of both the donor kidney and the islets), and (ii) encapsulation (or other physical isolation) of the islets to protect them from the recipient immune system without the need for immunosuppressive therapy.

## Research in Sweden

We must commemorate the late Carl-Gustav Groth, who, among his many contributions to kidney, pancreas, liver, and bone marrow allotransplantation in Sweden and globally [1], was the first to conduct clinical studies of porcine islet xenotransplantation. In 1994, Groth and his team at the Karolinska Institute undertook the xenotransplantation of wild-type fetal porcine islet-like cell clusters into insulin-dependent Type 1 diabetic kidney-transplant recipients [2]. The porcine cells were transplanted via the intraportal route and showed evidence of survival of some of them in the patients for several months. Urinary C-peptide, a marker of insulin production, was detected in some of the recipients. Groth’s team used the renal subcapsular route of islet delivery in two recipients. (The renal subcapsular space has subsequently been explored for transplantation of combined “islet-kidneys.”) Although none of the patients in Groth’s original clinical study achieved normoglycemia (or even a reduced insulin requirement), his contribution was important in encouraging others to advance islet xenotransplantation towards the clinic.

The Swedish study served as a catalyst for multiple clinical trials of islet xenotransplantation conducted outside the EU, as detailed by Piemonti et al. (2024) [3]. In 2000 in New Zealand Elliot and his team transplanted non-genetically modified (wild-type) encapsulated neonatal pig islets into two patients. The study showed improved glucose metabolism while addressing concerns about porcine endogenous retroviruses (PERVs). In 2005 Valdes led a study in Mexico involving transplantation of wild-type neonatal pig islets combined with Sertoli cells in a collagen-covered device and no immunosuppression into 12 patients. Improved glucose metabolic control was reported. By 2011, Matsumoto and colleagues in New Zealand reported on the transplantation of encapsulated wild-type neonatal pig islets intraperitoneally into 14 patients in the absence of immunosuppression. The study demonstrated reduced hypoglycemic unawareness. During the same year, Wang’s team in China transplanted neonatal pig islets from piglets negative for PERV-C into 21 patients via the intrahepatic route under immunosuppression. The results included reduced insulin requirements and improved HbA1c levels. In 2016, Matsumoto conducted another study in Argentina with eight patients. Neonatal pig islets (wild-type) were encapsulated and transplanted intraperitoneally without immunosuppression. This study demonstrated improved HbA1c and reduced hypoglycemic unawareness.

In 2018, Wang in China designed a clinical trial in accordance with Changsha regulations. Twenty patients received neonatal pig islets via the intraportal route, along with autologous T-regulatory cells and immunosuppression. The trial was successfully completed, highlighting advancements in immune regulation strategies to improve outcomes (unpublished).

Other Swedish researchers made major contributions to our knowledge of this field. Olle Korsgren’s work at Uppsala University over many years on porcine islet biology and transplantation has been pivotal in advancing the field of islet xenotransplantation, and he played a key role in the design and implementation of Groth’s pioneering study.

In subsequent studies with William Bennet, Korsgren identified a critical incompatibility between human blood and isolated islets, known as the instant blood-mediated inflammatory reaction (IBMIR), which significantly impacts the success of intraportal islet transplantation. They observed that both human and pig islets are damaged upon contact with human blood *in vitro* or when transplanted into the liver via the portal vein in nonhuman primates (NHPs). By dissecting the mechanisms behind the IBMIR, the Swedish team demonstrated that complement inhibitors and anticoagulants can reduce the damage to the islets [4, 5]. These findings contributed to a better understanding and modulation of IBMIR in the clinical setting of islet allotransplantation, helping improve the outcomes of both allo- and xeno-transplantation [6].

## Research in Germany

Significant contributions to the field of porcine islet xenotransplantation were made by members of the Justus-Liebig-Universität in Giessen, particularly in the areas of porcine islet isolation, preservation, and transplantation. The Giessen team, led by Reinhard Bretzel, developed methods to enhance the survival and function of porcine islets during cold storage and after transplantation into recipient animals [7]. Key advances by this team also improved the enzymatic digestion of the pancreas and the purification of porcine islets, along with refining the culture conditions that support the long-term functionality of the transplanted islets [8].

Moreover, the Giessen team shed light on the critical islet mass required to achieve successful metabolic function after transplantation in pig models [9]. These advances were crucial for increasing the number of islets available for experimental and preclinical studies, helping to make xenotransplantation more feasible.

Influenced by their work in Giessen, Bernhard Hering and Daniel Brandhorst moved from Germany to the USA and the UK, respectively, and further explored porcine islet isolation and transplantation and became prominent figures in the field of islet allo- and xeno-transplantation [10–13].

In Dresden, Germany, the group led by Barbara Ludwig has worked to optimize islet encapsulation technology. Their research uses a variety of animal models, including the isolation of mouse, rat, pig, and human islets. Using a novel immuno-protective membrane, the group demonstrated restoration of normoglycemia, without the need for immunosuppression, in a model of rat islet xenotransplantation into diabetic minipigs [14]. Ludwig’s team then showed success in NHP diabetic model, using porcine islet xenografts within a bioartificial pancreas device to create a suitable microenvironment for cell survival [15, 16]. Their BetaO2 device consists of islet compartments and a replenishable oxygen reservoir: a method which allowed better oxygenation and function of the islets, without immunological communication or pathogen transfer between the recipient and the donor graft. Ludwig and her researchers have taken steps towards safe clinical trials for patients with brittle diabetes to receive islet xenografts and continue to improve techniques in islet isolation and transplantation.

Other German groups have also contributed to investigations of porcine islet xenotransplantation by genetic engineering to reduce immune cell activation [17, 18].

## Research in Belgium

Under the leadership of Daniel Pipeleers at the Vrije Universiteit in Brussels, Marika Bogdani and colleagues investigated the growth and functional maturation of beta cells derived from endocrine implants of the prenatal porcine pancreas. Their research demonstrated that purified endocrine cells from the prenatal porcine pancreas could be implanted into hosts, successfully developing into mature, functional beta cells. This study highlights the critical importance of purifying endocrine cells from the prenatal porcine pancreas for successful transplantation [19].

At the Catholic University of Louvain, the work by Pierre Gianello and his team has significantly advanced the field of porcine islet xenotransplantation, particularly in improving islet isolation, addressing immune rejection, and exploring novel strategies like encapsulation. Denis Dufrane and Gianello demonstrated the survival of macro-encapsulated pig islets in NHPs without immunosuppressive therapy [20, 21]. Their patented alginate-coated collagen matrix device prevented entry of anti-pig antibodies, and allowed the islets to survive for up to 6 months. They refined critical factors, such as donor selection, isolation techniques, enzymatic digestion, and culture conditions, all of which enhance islet yield, viability, and functionality, making them more suitable for potential therapeutic use [20–22]. One of their innovative approaches relating to islet encapsulation has been successfully tested in preclinical studies involving NHPs, where they demonstrated a long duration of islet function, and aimed to optimize transplantation protocols for future clinical applications [23].

As with other areas of xenotransplantation, gene-modification strategies have also extended the limits of islet xenografts. Many European groups have delved into the development of the perfect gene-edited pig to yield the most suitable porcine islets. Pierre Gianello and Nizar Mourad published the first report of tailoring genetic modifications to increase insulin secretion from porcine islets [24, 25]. By modifying glucagon-like peptide-1 and type 3 muscarinic receptors in the pig, they were able to amplify glucose-stimulated insulin secretion [24]. With the important collaboration of Cesare Galli (Italy) (see below), they have now produced genetically-engineered pigs that produce significantly more insulin than any other pigs ([26], and see Mourad in this issue).

In a broader approach, Mourad and Gianello have also assessed the secretion of other pancreatic hormones, such as glucagon, noting both physiological similarities and challenges in matching human islet performance [26].

## Cesare Galli (Italy) – Gene-Edited Pigs

Cesare Galli is a prominent Italian researcher known for his groundbreaking work with gene-edited animals. He has over three decades of experience in assisted reproduction and biotechnology. He was actively involved in the development of nuclear transfer technology which led to significant milestones, such as cloning the progeny-tested bull Galileo in 1999, the first cloned horse, Prometea, in 2003, and the first cloned champion horse, Pieraz, in 2005 [27–29]. His team also successfully cloned piglets in 2006 and produced genetically-engineered pigs in 2008 by somatic cell nuclear transfer (SCNT) [30]. In 2009, he founded Avantea, which is internationally renowned in animal cloning. Galli’s use of CRISPR-Cas9 technology has enabled the creation of pigs lacking the Gal and Neu5Gc xenoantigens but transgenically expressing human “protective” proteins, including CD46, CD55, and CD59, crucial for reducing hyperacute rejection in xenotransplantation [31]. In 2014, Galli co-founded Xenothera SAS to further explore the biomedical applications of gene-edited pigs.

## Contributions From Europeans in the United States

### Research at the University of Minnesota

At the invitation of the well-known pancreas transplant surgeon, David Sutherland, Hering moved to the University of Minnesota, where he established a clinical islet allotransplantation program and experimental islet allotransplantation and pig islet xenotransplantation programs. By administering immunosuppressive therapy based on CD40/CD154 co-stimulation blockade [initially introduced into xenotransplantation research by Leo Buhler (Switzerland) and his colleagues in 2000], Hering’s group was among the first to report prolonged survival of diabetic NHPs after wild-type pig islet transplantation [10, 32].

The University of Minnesota raised funding to build a new “clean” (i.e., a designated pathogen-free) pig facility to enable clinical trials of pig islet xenotransplantation to be initiated, and the late Hendrik-Jan Schuurman (Netherlands) was brought in to establish this facility (Spring Point), which he did successfully. However, to our knowledge, no clinical trial of porcine islet xenotransplantation has yet taken place.

In addition to his contributions to experimental islet xenotransplantation, Hering has actively participated in defining the role and objectives of the International Xenotransplantation Association in regard to islet xenotransplantation [33, 34].

### Research at the University of Pittsburgh

In addition to Bernhard Hering and Hendrik-Jan Schuurman, other Europeans have worked in the USA either temporarily or permanently and have contributed significantly to progress in pig islet xenotransplantation. For several years, the Pittsburgh team included several research fellows from Jan Ijzerman’s transplant center at Erasmus University in Rotterdam. The islet transplantation program, led by Rita Bottino (Italy) with strong support from Massimo Trucco (Italy), and David KC Cooper (Great Britain) made extensive efforts to advance our understanding of xenotransplantation and addressed key challenges in the field.

#### The Induction of Diabetes

The optimization of a safe and reliable model for inducing diabetes in cynomolgus monkeys, as achieved by Pleunie Rood (Netherlands), represented a significant advance in diabetes research [35]. This model utilizes clinical-grade streptozotocin, a drug known to target and destroy islet beta cells, leading to insulin deficiency, stable elevation of blood glucose levels, and the establishment of a diabetic state over time.

Interestingly, Nagaraju and Bottino observed that streptozotocin administration induced transient but substantial lymphopenia, accompanied by an increase in absolute monocyte count and serum monocyte chemoattractant protein-1 (MCP-1). These contrasting effects can influence immune responses following islet transplantation [36].

A deeper understanding of the systemic effects of streptozotocin in the early period after administration, combined with enhanced knowledge of glucose regulation in NHPs - particularly cynomolgus monkeys - has significantly reduced stress and improved safety, which is crucial for ethical considerations.

#### Glucose Regulation in Genetically Engineered Pigs and Metabolic Compatibility Across Species

Advances in managing glucose regulation before and after transplantation in NHPs receiving porcine islets were highlighted by Anna Casu (Italy) and her colleagues [37]. They investigated glucose metabolic responses in both pigs and NHPs and discussed their implications for clinical translation. Casu further examined differences in insulin secretion and glucose metabolism between α1,3-galactosyltransferase gene-knockout (GTKO) pigs and wild-type pigs [38]. GTKO pigs showed differences in insulin secretion compared to wild-type pigs. Although the genetic or molecular basis for these differences was not determined, the study concluded that these physiological variations are unlikely to significantly impact the outcome of GTKO porcine islet xenotransplantation.

In subsequent research, Martin Wijkstrom (Sweden), who had spent time working with Bernhard Hering in Minnesota, studied glucose metabolism in genetically-modified pigs expressing human transgenic proteins regulated by an insulin promoter [39]. These transgenic pigs exhibited improved blood glucose regulation compared to wild-type pigs, suggesting that human gene expression under an insulin promoter enhances the potential of these pigs as sources of islets for xenotransplantation.

#### Loss of Islets From the IBMIR

As described initially by Bennet and his colleagues (see above), substantial islet loss often occurs early after their intraportal transplantation due to the IBMIR. Various strategies have been explored to mitigate these effects, including the administration of biologics (e.g., cobra venom factor) and pharmaceuticals (e.g., dextran sulfate) that inhibit complement activation, modulate coagulation, and reduce inflammation. Additionally, transgenic expression of proteins in donor pig islets that provide some protection from human complement and coagulation activation has proven effective in reducing the effects of IBMIR [40, 41].

**FIGURE 1:**
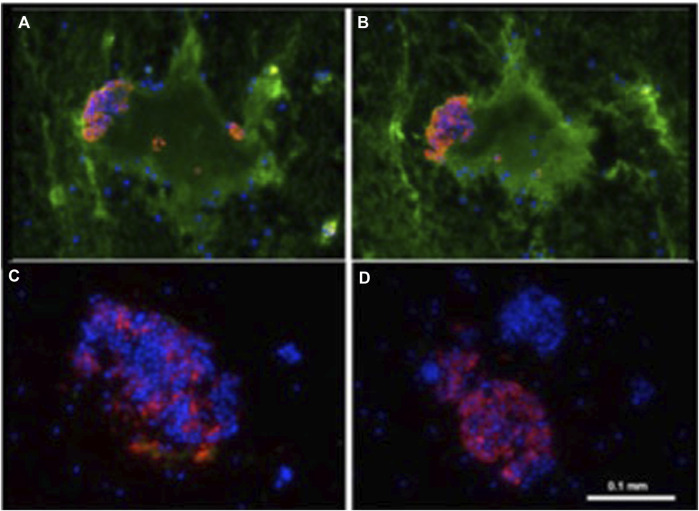
Binding of human IgM and IgG antibody to pig islets (xenogeneic) **(A, B)** and to human islets (allogeneic) **(C, D)**. IgM [green, **(A, C)**], IgG [green, (B, D)], insulin (red), nucleus (DAPI/blue). Yellow indicates colocalization of insulin and IgM/IgG. Adapted with permission from “Binding of human IgM and IgG antibody to pig islets (xenogeneic) **(A–B)** and to human islets (allogeneic) **(E–F)**. Binding of pig IgM and IgG antibodies to pig islets (autologous) **(C–D)**. IgM (green, A, C, E), IgG (green, B, D, F), insulin (red), nucleus (DAPI/blue). Yellow indicates colocalization of insulin and IgM/IgG” by Dirk J. Van Der Windt, Marco Marigliano, Jing He, Tatyana V. Votyakova, Gabriel J. Echeverri, Burcin Ekser, David Ayares, Fadi G. Lakkis, David K. C. Cooper, Massimo Trucco and Rita Bottino, licensed under CC BY-NC.

In an *in vitro* model of IBMIR, Dirk van der Windt (Netherlands) investigated the mechanisms underlying early islet graft damage and found that humoral immunity played a more important role in triggering and exacerbating IBMIR than previously recognized (Figure 1) [42]. In similar settings, using neonatal islet-like cell clusters from wild-type and GTKO pigs expressing human CD46 (GTKO/CD46) or CD46 and CD39 (GTKO/CD46/CD39), Nagaraju et al. demonstrated that neonatal islet-like cell clusters experienced humoral injury regardless of their genetic modifications [43].

#### Intraportal Adult Pig-To-NHP Islet Transplantation

**FIGURE 2:**
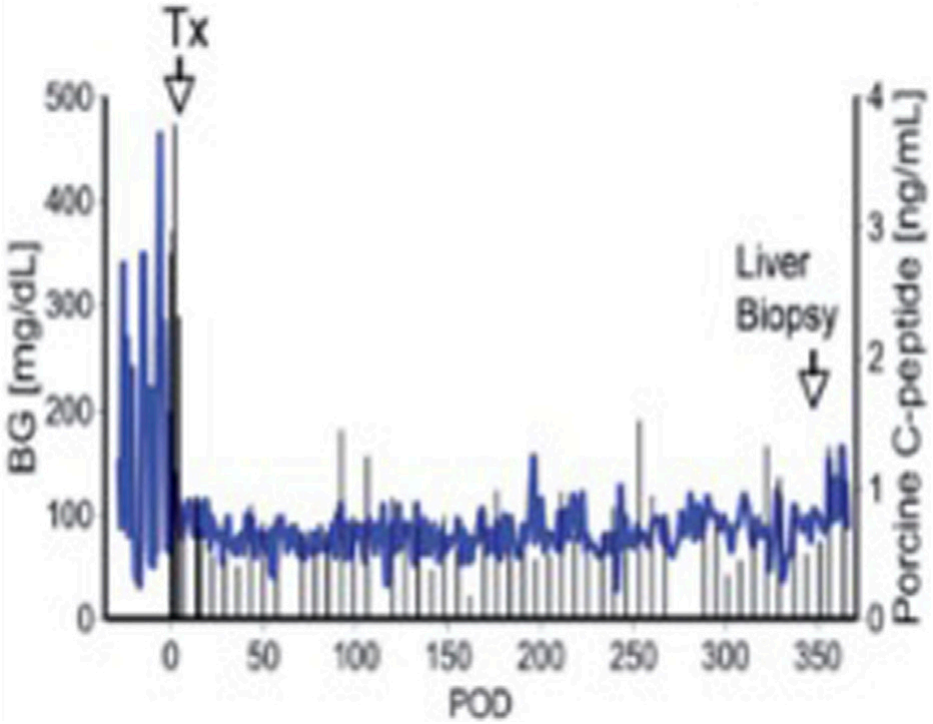
Blood glucose and pig C-peptide levels in a streptozotocin-induced diabetic cynomolgus monkey before and after intraportal transplantation of islets from a pig expressing the human complement-regulatory protein, CD46. No exogenous insulin was administered after the transplant. The normoglycemic monkey was electively euthanized after 12 months. Day 0 = day of islet transplantation. Adapted with permission from “Blood glucose and pig C-peptide levels in a streptozotocin-induced diabetic cynomolgus monkey before and after intraportal transplantation of islets from a pig expressing the human complement-regulatory protein, CD46. No exogenous insulin was administered after the transplant. The normoglycemic monkey was electively euthanized after 12 months. Day 0 = day of islet transplantation” by Cooper D. K.C. and Bottino R., licensed under CC BY-NC-SA 4.0.

Successful long-term survival of islet grafts was achieved by Dirk van der Windt and his colleagues through a combination of strategies, including optimized diabetes induction in cynomolgus monkeys, an anti-inflammatory protocol, an immunosuppressive regimen based on an anti-CD154 monoclonal antibody (mAb), and the use of pig islets expressing the human complement regulator CD46 [44]. This was the first report of pig islet transplantation resulting in insulin-independence and normoglycemia for a period of >12 months (Figure 2). This significant achievement was later replicated using GTKO pig donors that expressed multiple human transgenes targeting complement and coagulation activation and inflammation [46]. It demonstrated the beneficial effects of tissue targeted gene editing in modulating IBMIR and early islet loss, thus advancing the acceptance of genetically-engineered porcine tissues for transplantation. However, in neither study were the results consistently successful.

Conducting pre-clinical trials with long-term follow-up enabled the refinement of methodologies for monitoring diabetes in NHPs post-transplantation. Marco Marigliano (Italy) and his colleagues demonstrated that monitoring hemoglobin A1C is a reliable metric for assessing glucose control both before and after islet xenotransplantation [47].

Standardization of experimental conditions ensured the overall safety of the procedure. However, complications were reported. For example, Nathalie Campanile (Switzerland) reported that, 1 week after intraportal pig islet transplantation, one recipient developed acute gastric dilatation that proved fatal [48]. Acute gastric dilatation had been described before in NHPs and has subsequently proved problematic in baboons after pig organ transplantation. The report by the Pittsburgh group offered guidelines for preventing or managing acute gastric dilatation.

Rita Bottino and Angela Criscimanna (Italy) reported interesting findings related to the recovery of beta cell mass and function in NHPs that had been rendered diabetic with streptozotocin and subsequently received porcine islet transplants [49]. Although the return of native beta cell function following the induction of hyperglycemia and islet transplantation in large animal models is rare, it can be a significant confounding factor if not properly detected, potentially impacting the interpretation of islet graft performance. The recovery of native beta cell function underscored the need for mechanistic investigations to explore the regenerative properties of pancreatic cells and the potential role of transplantation. It also highlighted the importance of rigorous monitoring of islet graft performance and the selection of testing methods that can distinguish between endogenous and donor islet metabolic responses.

#### Exploration of Immunosuppressive Agents in NHPs

Alemtuzumab (Campath 1H) is a potent immunosuppressive antibody that targets CD52, a molecule expressed on lymphocytes in humans and some NHPs. However, in many NHPs CD52 is also expressed on erythrocytes which are hemolyzed after the administration of alemtuzumab. A small subset of cynomolgus moneys (from Indonesia) do not express CD52 on their erythrocytes and are, therefore, suitable for studies of alemtuzumab.

Although not specific to porcine islet xenotransplantation, Dirk van der Windt (working with Fadi Lakkis) investigated the effects of alemtuzumab on lymphocyte depletion and subsequent repopulation in Indonesian cynomolgus monkeys [50]. Alemtuzumab effectively depleted all circulating lymphocytes in the monkeys. This depletion was both rapid and profound – more profound than after the administration of anti-thymocyte globulin - impacting both T and B cells. Lymphocyte repopulation occurred gradually over time, the repopulation patterns varying among different lymphocyte subsets, with some recovering more quickly than others. The study provided valuable insights into the kinetics of lymphocyte depletion and repopulation.

It was well-known that the anti-CD154mAbs available more than 10 years ago were thrombogenic in NHPs with organ allografts or xenografts [51–53]. However, after pig islet transplantation, the administration of an anti-CD154mAb was found to be both effective and safe [54]. While trials in humans initially identified thromboembolic events as a safety concern, these were not clinically or microscopically observed in a study of >20 cynomolgus monkeys receiving pig islet grafts. No evidence of thrombosis or thromboembolism was documented despite careful histopathological examination, including of the brain. It was hypothesized that the exposure of the recipient to the very small antigen load of islets (when compared with a whole organ) may have been insufficient to induce thrombosis.

Furthermore, no other adverse effects were reported. These findings supported the use of anti-CD154mAb as a promising strategy for enhancing the survival and function of pig islet transplants in NHPs, potentially warranting reconsideration for human use. However, the subsequent introduction of Fc-modified anti-CD154mAbs that have been designed to prevent any thrombogenic effect has ensured their safety in this respect.

#### The Gastric Submucosal Space (GSMS) as an Alternative Site for Islet Transplantation

As a preliminary for studies in the pig-to-NHP model, the GSMS was explored as a site for pig islet allotransplantation. The GSMS is an accessible location and has a rich vascular supply and may have potential advantages over the traditional intraportal site for islet transplantation. Gabriel Echeverri and colleagues developed a technique for transplanting islets into the GSMS using endoscopy [55]. This approach demonstrated potential for reversing diabetes in diabetic pigs, as evidenced by reduced blood glucose levels. Further studies by Fujita and Tanaka confirmed the feasibility of obtaining endoscopic biopsies from this site and also demonstrated that these biopsies allowed for detailed histological examination [56, 57]. However, in a limited clinical trial by James Shapiro (UK and Canada) and his colleagues, the GSMS was found to be less successful than hoped [58].

## Pig Neural Cell Xenotransplantation in the Treatment of Parkinson’s Disease

In an effort to investigate whether pig neural cell precursors could prove to be a possible treatment option for neurodegenerative disorders, Aron Badin, Cozzi, Hantraye, and their colleagues conducted very interesting studies in NHPs with a pharmacologically-induced Parkinson disease-like condition [59]. They transplanted into immunosuppressed Parkinsonian monkeys, neural precursors from either wild type or genetically-engineered pigs in which fetal mesencephalic neurons expressed CTLA4-Ig.

The results obtained in these studies were impressive. NHPs with xenografts showed an extraordinary recovery of locomotor function that persisted for as long as 31 months in one recipient of a CTLA4-Ig porcine embryonic graft. Furthermore, such a clinical recovery was associated with restoration of dopaminergic activity as demonstrated by positron emission tomography and histological examination. In addition, these studies demonstrated that, notwithstanding the brain is considered an immuno-privileged site, at least in this model systemic immunosuppression appeared indispensable to achieve long-term neural xenograft survival.

Unfortunately, these exciting and encouraging studies had to be discontinued when certain countries in Europe banned research involving NHPs.

## Xenotransplantation of Bone Marrow Cells and Hepatocytes

Leo Bühler (Switzerland) has dedicated much of his career to advancing the clinical feasibility of xenotransplantation and overcoming its associated immunological challenges. His contributions span multiple aspects of the field, including hematopoietic cell, islet, and hepatocyte xenotransplantation, as well as developing frameworks for safe and ethical xenotransplantation clinical trials.

Bühler’s early studies focused on hematopoietic cell transplantation in the pig-to-nonhuman primate (NHP) model [60]. It was when studying this challenging problem that he first demonstrated that co-stimulation blockade of the CD40/CD154 pathway provided better immunosuppression than conventional therapy. These investigations emphasized the importance of using appropriate anti-rejection, antithrombotic, and anti-inflammatory agents to address complications [61, 62].

His research subsequently extended to hepatocyte transplantation, where he explored innovative methods to treat acute liver failure through xenotransplantation [63, 64]. His team investigated the transplantation of encapsulated hepatocytes in both mice and baboons, revealing survival benefits without stimulating native liver regeneration. Further work included the development of strategies of co-encapsulation of hepatocytes with mesenchymal stromal cells (MSCs) to improve porcine hepatocyte viability and functionality [65].

Leo has also made major contributions to the field of xenotransplantation by (i) his long-term role as editor of the official journal of the International Xenotransplantation Association (IXA) and (ii) by maintaining a registry of the clinical transplants carried out in the world [66].

Table 1 summarizes the significant contributions detailed above.

**TABLE 1:** Summary of key contributions to cell and islet xenotransplantation research by country and researcher/team.

Country	Researcher/Team	Contributions
Sweden	Carl-Gustav Groth	Conducted first clinical studies on porcine islet xenotransplantation; developed intraportal and renal subcapsular transplantation techniques [1, 2]
	Olle Korsgren, William Bennet	Identified IBMIR; studied porcine islet biology; developed complement inhibitors and anticoagulants to mitigate IBMIR damage [4, 6]
Germany	Reinhard Bretzel Daniel Brandhorst	Improved porcine islet isolation, enzymatic digestion, and culture; studied critical islet mass for transplantation success [7–12]
	Barbara Ludwig	Developed islet encapsulation technologies; demonstrated normoglycemia restoration using bioartificial pancreas devices [14–16]
Belgium	Daniel Pipeleers Marika Bogdani	Explored the functionality of porcine islets for xenotransplantation, insulin secretion dynamics and compatibility with human systems [19]
	Pierre Gianello Denis Dufrane Nizar Mourad	Improved islet isolation, encapsulation, and viability; developed alginate-coated collagen matrix for macro-encapsulation; enhanced genetic modifications for insulin secretion [20–26, 67]
Italy	Cesare Galli	Pioneered gene-edited pigs using CRISPR-Cas9; developed pigs expressing human complement regulators to reduce hyperacute rejection [27–31]
	Emanuele Cozzi	Explored neural precursor transplantation for Parkinson’s treatment; demonstrated long-term recovery of locomotor function in NHP models [59]
USA (Europeans)	Bernhard Hering (Germany)	Advanced islet allotransplantation and xenotransplantation; developed clean pig facilities; studied anti-CD154 mAb immunosuppressive strategies [10–13, 32–34]
	David KC Cooper (Great Britain) Rita Bottino (Italy)	Investigated beta-cell recovery and IBMIR effects; developed diabetes models in NHPs; optimized transplantation techniques [35, 36, 46, 49]
	Dirk van der Windt (Netherlands)	Explored IBMIR mechanisms and mitigation strategies; achieved long-term survival of pig islet transplants in NHPs [41, 42, 44, 50]
	Marco Marigliano (Italy)	Standardized hemoglobin A1C monitoring for glucose control in preclinical transplantation studies [47]
	Anna Casu (Italy)	Investigated glucose metabolism differences between genetically modified and wild-type pigs and intraspecies compatibility [37, 38]
	Martin Wijkstrom (Sweden)	Researched glucose metabolism in genetically modified pigs expressing human transgenes regulated by insulin promoters [39]
	Nathalie Campanile (Switzerland)	Reported complications of intraportal porcine islet transplantation in NHP [48]
	Pleunie Rood (Netherlands)	Achieved safer induction of diabetes in NHP and contributed to reduce islet graft loss following intraportal porcine islet transplantation [35, 40]
Netherlands	Henk Shuurman	Contributed to advancement in ethical, regulatory, and preclinical aspects of xenotransplantation [10, 13]
Switzerland	Leo Buhler	Explored hematopoietic and hepatocyte xenotransplantation; developed immunological strategies for clinical trials [60–66]

## Comment

As with our accompanying review of the contributions of Europeans to research into pig organ xenotransplantation, space has not allowed us to draw attention to all of the Europeans who have worked on islet or cell xenotransplantation, and we apologize for our omissions. We also apologize to the many non-Europeans who have contributed significantly to the research reviewed in this article but who have not been identified. The advances we have reviewed provide evidence for the contributions of European surgeons and scientists, in collaboration with their non-European colleagues, to the potentially important field of islet xenotransplantation.

## Abbreviations

GSMS, gastric submucosal space; GTKO, α1,3-galactosyltransferase gene-knockout; IBMIR, instant blood-mediated inflammatory reaction; mAb, monoclonal antibody; NHP, nonhuman primate.
